# A Fiber-Based Chromatic Dispersion Probe for Simultaneous Measurement of *X*-Axis and *Z*-Axis Displacements with Nanometric Resolutions

**DOI:** 10.3390/s23010051

**Published:** 2022-12-21

**Authors:** Ran Zhao, Chong Chen, Xin Xiong, Yuan-Liu Chen, Bing-Feng Ju

**Affiliations:** 1 The State Key Laboratory of Fluid Power and Mechatronic Systems, Zhejiang University, Hangzhou 310058, China; 2 Hangzhou Global Scientific and Technological Innovation Center, Zhejiang University, Hangzhou 310027, China

**Keywords:** fiber-based probe, chromatic dispersion, full width at half maxima, centroid wavelength, simultaneous displacement measurement, nanometric resolution

## Abstract

In this paper, a fiber-based chromatic dispersion probe for simultaneous measurement of *X*-axis and *Z*-axis displacements with nanometric resolutions by using the full width at half maxima (FWHM) of the detected spectral signal has been proposed and demonstrated. For *X*-axis, FWHM is employed for indicating the *X*-axis displacement based on the fact that the FWHM remains almost constant with the varying *Z*-axis displacement of the fiber detector and shows a linear relationship with the *X*-axis displacement within a specific *Z*-axis displacement range. For the *Z*-axis, the linear relationship between the centroid wavelength *λ* of the detected spectral signal and the *Z*-axis displacement is employed for indicating the *Z*-axis displacement based on the fact that the sensitivity (slope of the *λ-Z* curve) is also linear with *X*-axis displacement within a certain *X*-axis displacement range. Theoretical and experimental investigations have verified the feasibility of the proposed chromatic dispersion probe, which yields *X*- and *Z*-axis measurement ranges of 2.3 μm and 15 μm and *X*- and *Z*-axis measurement resolutions of better than 25 nm and 50 nm, respectively. Experiments were further performed to evaluate the basic performance of the prototype probe and the maximum measurement errors were less than 10 nm and 60 nm for *X*- and *Z*-axis displacements, respectively.

## 1. Introduction

With the miniaturization of equipment and components in the industry, microscale stages have been a powerful tool and widely used in the space-limited and small-cavity fields of micro-electro-mechanical systems (MEMS) [1,2,3], precision optics [4,5,6], microscale mechanical testing [7], semiconductor manufacturing [8], and so on. To ensure the high-accuracy motion of these microscale stages, high-precision, cost-effective, and easy-mounted displacement measurement sensors are necessary and needed [9,10,11].

To address the issue of the displacement measurement of the micro-stage and fulfill the development of the application of the displacement measurement in the space-limited and small-cavity fields, many displacement measurement sensors and techniques [11,12,13,14,15,16,17,18,19] have been proposed so far, such as optical interferometers [11,12,13], digital image correlation sensors [14,15], thin film displacement sensors [16,17], integrated capacitive displacement sensors [18,19], hall-effect sensors [20], and so on. For the optical interferometers, not only the miniature laser diode interferometer [11], the compact laser interferometer based on the displacement sensing method [12], but also the Fabry–Pérot interferometer [13] can achieve good measurement resolutions with large-stroke measurement ranges. However, due to the large-stroke optical path, these optical interferometers are relatively fragile and sensitive to the environmental disturbances of temperature, humidity, pressure, as well as air turbulence, which will change the air refractive index beyond that it is complicated to be mounted [21,22]. More importantly, it is difficult to eliminate the reference mirror error of the optical interferometer [22,23]. The digital image correlation sensors are also proposed for the displacement measurement of the small-sized objects but complex optical components and auxiliary equipment narrow their practical application in industry [24]. As for the thin film displacement sensors [16,17], the integrated capacitive displacement sensors [18,19], and the hall-effect sensors [20], they can also realize good performances for displacement measurement of the micro-stages. Nevertheless, because of the electronic components within these sensors, electrical and electromagnetic interference are the main demerits of these sensors [25,26].

On the other hand, with the development of fiber optics, optical fiber has been widely used in the displacement measurement sensors for the measurement of various microscale specimens with its remarkable features of immunization to electrical and electromagnetic disturbance, geometric flexibility, corrosion resistance, and easy-mounted property together with portability [27,28]. Optical fiber-based interferometers have been developed for the displacement measurement of single-axis micro-stages [29,30]. A differential fiber-based displacement sensor has also been established for the displacement measurement of a one-axis micro-stage [31]. Based on the macro-bending effect, optical fiber-based sensors have been constructed for the displacement measurement of the dual-axis of the measured micro-stage [29,32]. However, in the above fiber-based sensors, most of the sensors can only measure one axis displacement, and extra optical components are necessary if more than one axis needs to be measured, which will lead to more errors introduced to the sensors and the complexity of the overall systems [12,33]. As for the optical fiber-based sensors based on the macro-bending effect, these sensors have shown good sensitivity and stability, but because of the core-cladding modal coupling, the detected spectral signals will experience wavelength-dependent losses [34]. A fiber-based and cost-effective displacement sensor that can measure dual-axis displacement simultaneously is, hence, one of the key points to expand the practical application of displacement measurement in the space-limited and small-cavity fields in the industry. To realize the simultaneous measurement of the dual-axis displacement by a single fiber-based displacement sensor, another spectral parameter should be used as the displacement measurement indicator. The FWHM of the detected spectral signal is a widely employed spectral parameter for the evaluation of the measurement resolution [35], the stress distribution monitoring [36], as well as the measurement of the microscope’s sectioning power [37] with its easy understanding and flexibly obtained properties. The FWHM of the detected spectral signal can, thus, be a powerful potential tool for dual-axis displacement measurement, which is almost unreported to our knowledge.

Therefore, in this paper, a fiber-based cost-effective chromatic dispersion probe for simultaneous measurement of *X*-axis and *Z*-axis displacements with nanometric resolutions by using the FWHM of the detected spectral signal has been proposed. For *X*-axis, FWHM is employed for indicating the *X*-axis displacement based on the fact that FWHM remains constant with the varying *Z*-axis displacement of the fiber detector and shows a linear relationship with the *X*-axis displacement within a specific *Z*-axis displacement range. For the *Z*-axis, the linear relationship between the centroid wavelength *λ* of the detected spectral signal and the *Z*-axis displacement is employed for indicating the *Z*-axis displacement based on the fact that the sensitivity (slope of the *λ-Z* curve) is also linear with the *X*-axis displacement within a certain *X*-axis displacement range. The basic performance of the prototype probe has been investigated and verified. It should be pointed out that due to the spectral instability caused by the influence of the light source stability, environmental temperature, and humidity, the center-of-mass method has been used in the process of calculating the centroid wavelength of the obtained normalized spectra from the fiber detector. Employment of the center-of-mass method to calculate the centroid wavelength can make ensure constant stable measurement results and can be regarded as a sign of the robustness of the proposed probe.

## 2. Principle and Simulation

**Figure 1:**
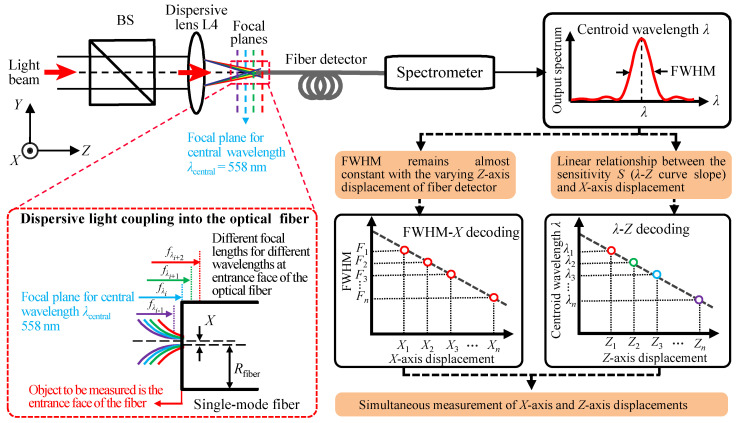
Schematic diagram of the established fiber-based chromatic dispersion probe for the simultaneous measurement of *X*-axis and *Z*-axis displacements with nanometric resolutions.

Figure 1 depicts the schematic diagram of the established fiber-based chromatic dispersion probe for simultaneous measurement of *X*-axis and *Z*-axis displacements in which the object to be measured is the entrance face of the employed optical single-mode fiber. By the detected output spectrum from the fiber detector, within the *X*-axis, FWHM of the output spectrum is employed for indicating the *X*-axis displacement based on the fact that FWHM remains almost constant with the varying *Z*-axis displacement of the fiber detector and shows a linear relationship with the *X*-axis displacement within a specific *Z*-axis displacement range. For the *Z*-axis, the linear relationship between the centroid wavelength *λ* of the detected spectral signal and the *Z*-axis displacement is employed for indicating the *Z*-axis displacement based on the fact that the sensitivity *S* (slope of the *λ-Z* curve) is also linear with the *X*-axis displacement within a certain *X*-axis displacement range. In the decoding process of the simultaneous measurement of *X*-axis and *Z*-axis displacements, firstly, the FWHM is calculated from the detected spectrum and then the displacement of the *X*-axis is decoded through the FWHM. Secondly, with the obtained *X*-axis displacement, the sensitivity *S* (slope of the *λ*-*Z* curve) by the *S-X* curve is decoded and obtained. Finally, by the decoded sensitivity *S*, the corresponding *λ*-*Z* curve can be selected and by the *λ-Z* curve, the *Z*-axis displacement can be approached. It should be pointed out that the above operations can only be realized within certain *X*- and *Z*-axis displacement ranges, which are associated with the measurement ranges of the *X*- and *Z*-axis of the established chromatic dispersion probe. It should also be noted that the dispersive light coupling into the entrance face of the optical fiber core has many continuous focal planes within the fiber detector of the established chromatic dispersion probe, and one of the focal planes shown in Figure 1 is the focal plane corresponding to the central wavelength of the light beam.

### 2.1. Chromatic Dispersion and Imaging Principle

**Figure 2:**
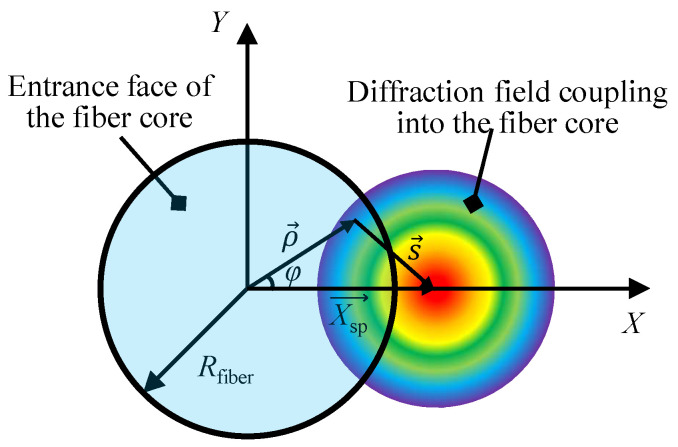
Schematic of the diffraction field coupling into the entrance face of the core of an optical fiber and the corresponding coordinate system in the entrance face of the fiber core.

In the established fiber-based chromatic dispersion probe, the light beam passing through a small pinhole within the 4*f* optical system filter can be regarded as the linear superposition of the paraxial point light sources, which can be simply signified as the superposition of the impulse functions of ∑*δ*(*r*_sp_). In the chromatic dispersion probe, an always coherent optical system is realized by employing the single-mode optical fiber as the signal-transferring media regardless of the detailed optical paths and the fiber dimensions [38,39]. The axial response of the chromatic dispersion probe within the entrance face of the fiber detector can thereby be expressed as:

(1) $$I(u,v)=|\sum_{r_{\mathrm{sp}}}h_{\mathrm{effective}}(u,v){|}^{2}.$$

where *r*_sp_ stands for the distance between the light source center and the superimposed paraxial point light source and *h*_effective_ is the effective point spread function of the probe system associated with the single-mode fiber and can be expressed as:

(2) $$h_{\mathrm{effective}}=h(u,v)⊗e(v),$$

where *h*(*u*, *v*) is the point spread function of the probe system, *e*(*v*) is the eigenfunction of the transverse orthonormal modal field in the entrance face of the single-mode fiber, ⊗ is the convolution operation, and *u* and *v* are the optical coordinates related to the *Z*-axis displacement *Z-* and the *X*-directional distance *X*_sp_. *u* and *v* can be expressed as:

(3) $$u=\frac{2\pi }{\lambda }Z\sin^{2}\alpha ,$$

(4) $$v=\frac{2\pi }{\lambda }(\vec{\rho }-{\vec{X}}_{\mathrm{sp}})\sin\alpha ,$$

where sin *α* is the numerical aperture (NA) of the employed chromatic dispersive lens, *ρ* is the radial variation of the incident field of the fiber shown in Figure 2, and the *X*- directional distance *X*_sp_ is the distance between the center of the fiber core and the diffraction field generated by the dispersive lens and can be expressed as the distance between the light source center and the superimposed paraxial point light sources *r*_sp_ and the *X*-axis displacement *X* given by Equation (5):

(5) $$X_{\mathrm{sp}}=\sqrt{{\left(r_{\mathrm{sp}}cos(\theta )/M+X\right)}^{2}+{\left(r_{\mathrm{sp}}\sin(\theta )/M\right)}^{2}}.$$

where *M* is the magnification of the optical system. In the simulation, *θ* is the angle corresponding to the discrete paraxial point light source that needs to be superimposed in a circle from the center of the light source *r*_sp_.

The point spread function for the established optical system is given as:

(6) $$h(u,v)=A\int_{0}^{1}e^{-\frac{ju}{2}r^{2}}J_{0}(vr)rdr.$$

where *J*_0_ represents the zero-order Bessel functions of the first kind.

It should be pointed out that the used modal field in Equation (2) is given by Ref. [40].

(7) $$\vec{e_{n}}=\hat{x}\frac{f_{n}(R)}{\sqrt{\psi_{n}}}\cos(n-1)\varphi ,$$

where *ψ_n_* is the normalization coefficient of the mode, *φ* is the azimuthal angle shown in Figure 2 and *f*_n_(*R*) represents the transverse electromagnetic field of the used step-index optical fiber and can be expressed as [40,41]:

(8) $$f_{n}(R)=\left\{\begin{array}{c} \frac{J_{n-1}(UR)}{J_{n-1}(U)},R\leq 1\\ \frac{K_{n-1}(WR)}{K_{n-1}(W)},R>1 \end{array}\right.,$$

with *R* = *ρ/R*_fiber_ and *U* is the modal eigenvalue [41], in which *U* and *W* are related to the dimensionless parameter *V* for the core and cladding of the fiber and can be found in Ref. [42] (p. 314). Moreover, *n* stands for the *n*_th_ mode of the employed fiber and equals 1 in the single-mode fiber.

(9) $$V^{2}=W^{2}+U^{2},$$

The effective point spread function can be expressed by bringing Equations (6) and (7) into Equation (2), which is given by:

(10) $$h_{\mathrm{effective}}=A\int_{0}^{\infty }\int_{0}^{1}e^{-\frac{ju}{2}r^{2}}J_{0}(vr)rdr\cdot f_{1}(R)RdR=A\int_{0}^{1}e^{-\frac{ju}{2}r^{2}}J_{0}(v_{\mathrm{diff}}r)r\times \int_{0}^{\infty }J_{0}(v_{c}rR)f_{1}(R)RdRdr,$$

where *v*_c_ represents the optical coordinate and is given by 2π *R*_fiber_ sin *α*/*λ* and *v*_diff_ is the optical coordinate of the diffraction field given by 2π *X*_sp_ sin *α*/*λ*.

The axial responses of the measurement axis can be expressed by bringing Equations (8)–(10) into Equation (1):

(11) $$I(u,v)={\left|\sum_{r_{\mathrm{sp}}}\frac{A\sqrt{\psi_{1}}}{2\pi R_{\mathrm{fiber}}}\int_{0}^{1}e^{-\frac{ju}{2}r^{2}}J_{0}(v_{\mathrm{diff}}r)\frac{V^{2}\left[rv_{c}J_{1}(rv_{c})-J_{0}(rv_{c})\frac{UJ_{1}(U)}{J_{0}(U)}\right]}{\left({\left(rv_{c}\right)}^{2}-U^{2}\right)\left({\left(rv_{c}\right)}^{2}+W^{2}\right)}rdr\right|}^{2}.$$

where *J*_1_(*rv*_c_) is the first-order Bessel function of the first kind.

### 2.2. Measurement Principle of the Simultaneous Measurement of X-Axis and Z-Axis Displacements

**Figure 3:**
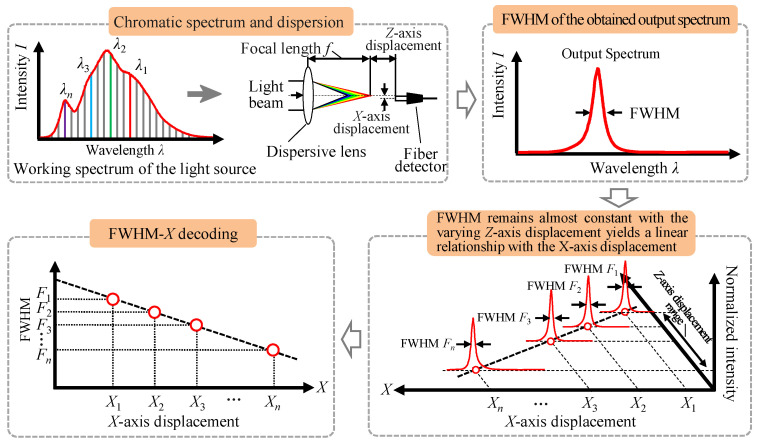
A flowchart of the measurement principle and the corresponding decoding process of the proposed fiber-based chromatic dispersion probe for the displacement measurement of the *X*-axis.

Figure 3 shows a flowchart of the measurement principle and the corresponding decoding process of the proposed fiber-based chromatic dispersion probe for displacement measurement of the *X*-axis. After passing through the optical path, the output spectrum is obtained and detected by the fiber detector in which the FWHM can be calculated. With different *X*-axis displacements of the fiber detector, different output spectra are acquired with different FWHM values. FWHM-*X* decoding can be carried out based on the fact that FWHM remains almost constant with the varying *Z*-axis displacement and yields a linear relationship with the *X*-axis displacement within a specific *Z*-axis displacement.

**Figure 4:**
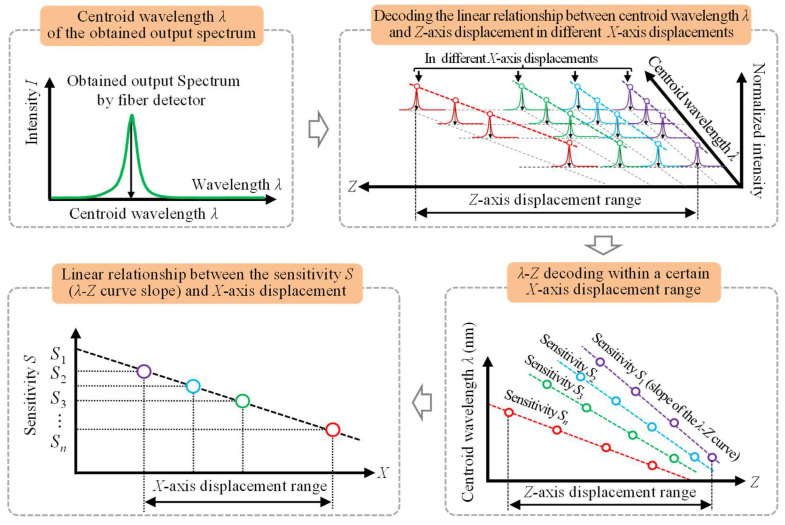
A flowchart of the measurement principle and the corresponding decoding process of the proposed fiber-based chromatic dispersion probe for displacement measurement of the *Z*-axis.

Figure 4 shows a flowchart of the measurement principle and the corresponding decoding process of the proposed fiber-based chromatic dispersion probe for displacement measurement of the *Z*-axis. Additionally, with the detected output spectrum, the centroid wavelength *λ* can be obtained. Thus, decoding the linear relationship between the centroid wavelength *λ* and *Z*-axis displacement in different *X*-axis displacements can be performed, in which different sensitivity *S* (*λ-Z* curve slopes) can be observed. With the different sensitivity *S* (*λ-Z* curve slopes), the linear relationship between the sensitivity *S* and *X*-axis displacement can also be inspected within a specific *X*-axis displacement range.

### 2.3. Simulation and Discussion

**Figure 5:**
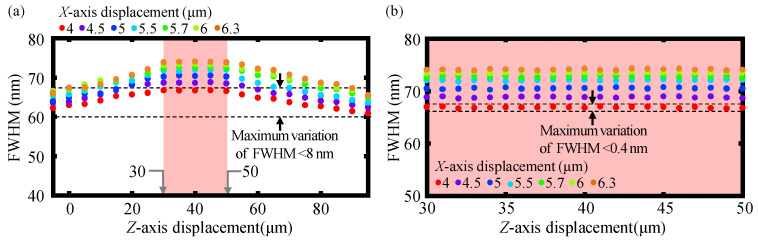
Simulation results of (**a**) the variation of FWHM within *Z*-axis displacement ranging from −5 to 95 μm; and (**b**) the corresponding *Z*-axis displacement ranging from 30 μm to 50 μm at the *X*-axis displacements from 4 μm to 6.3 μm.

According to Equation (11) in Section 2.1, the axial response curves corresponding to different *X*- and *Z*- axis displacements of the established chromatic dispersion probe are calculated and plotted by the software MATLAB. The FWHM of the axial response curves can, thereby, be obtained by finding the half-peak width of these axial response curves. It should be noted that the axial response curves have gradually become spectral bimodal distribution when the *X*-axis displacement is larger than two microns based on the theory of the employed step-index single-mode fiber, in which the cladding of the step-index single-mode fiber can hardly accept the dispersive light coupling into it. Meanwhile, in practical experiments, not only the core but also the cladding of the step-index single-mode fiber can receive the dispersive light coupling into it; moreover, the focused beam spot diameter by the dispersive lens L4 in the experiment is larger than that of the focused beam spot diameter in simulation due to the influence of the beam quality and the lens spherical aberration. Consequently, no spectral bimodal distribution is found in the practical experiments and Gaussian fitting is used for the calculation of the FWHM in the simulation. Therefore, there is a difference in the FWHM of the simulation and experimental results. Figure 5a,b show the simulation results of the variation of FWHM within the *Z*-axis displacement ranging from −5 to 95 μm and *Z*-axis displacement ranging from 30 to 50 μm at *X*-axis displacements from 4 to 6.3 μm. As depicted in the figures, the maximum variation of FWHM within the *Z*-axis displacement ranging from −5 to 95 μm is less than 8 nm and is even less than 0.4 nm within the corresponding ranging from 30 to 50 μm at the *X*-axis displacements ranging from 4 to 6.3 μm. It is observed that FWHM remains almost constant with the varying of the *Z*-axis displacement of the fiber detector within *Z*-axis displacement ranging from 30 to 50 μm and can be used for indicating the *X*-axis displacement regardless of the simultaneous variation of the *Z*-axis displacement.

**Figure 6:**
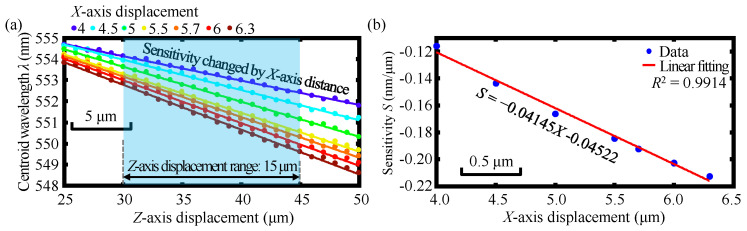
Simulation results of (**a**) the linear relationship between the centroid wavelength *λ* and *Z*-axis displacement and (**b**) the linear relationship between the corresponding sensitivity *S* (*λ*-*Z* curve slope) and *X*-axis displacement at the *X*-axis displacements from 4 to 6.3 μm.

Figure 6a,b show simulation results of the linear relationship between the centroid wavelength *λ* and *Z*-axis displacement and the linear relationship between the corresponding sensitivity *S* (*λ*-*Z* curve slope) and *X*-axis displacement at the *X*-axis displacements from 4 μm to 6.3 μm. As can be observed in the figures, the centroid wavelength *λ* of the detected spectral signal can be employed for indicating the *Z*-axis displacement associated with different *X*-axis displacements by using the corresponding linear decoding of the *S-X* curve within a specific *Z*-axis displacement range of 15 μm.

## 3. Experiments

Experiments on the investigation of the *X*- and *Z*-axis measurement ranges and resolutions were carried out first. Experiments were further performed to evaluate the basic performance of the prototype probe for simultaneous measurement of *X*-axis and *Z*-axis displacements with nanometric resolutions.

**Figure 7:**
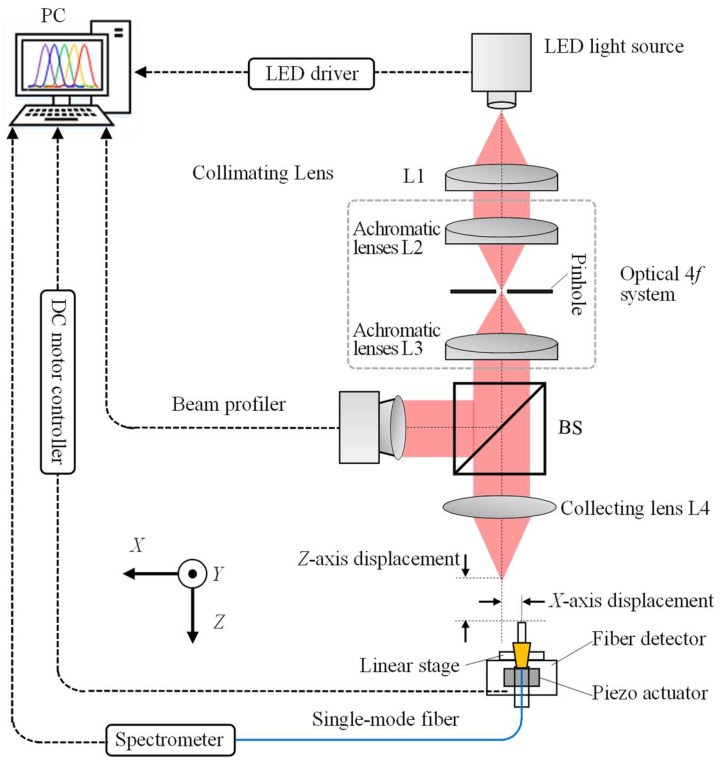
Schematic of the fiber-based chromatic dispersion probe for simultaneous measurement of the *X*-axis and *Z*-axis displacements with nanometric resolutions.

Figure 7 shows the schematic of the experimental configuration of the proposed chromatic dispersion probe for simultaneous measurement of the *X*- and *Z*-axis displacements with nanometric resolutions. To obtain a wide spectral range for measurement, a chromatic LED illuminator (L1CU-4090, Lumileds Holding B.V., Haarlemmermeer, The Netherlands) was selected as the light source with a band-wide spectrum ranging from 400 nm to 800 nm. Collimated by a collimating lens L1 (AC 254-030-A-ML, Thorlabs Inc., Newton, NJ, USA), the light beam was made to pass through an optical 4*f* system in which a pinhole with a diameter of 50 μm was placed onto the focal planes of the two achromatic lenses L2 and L3. After that, the light beam was split into two sub-beams called the reference sub-beam and the measurement sub-beam by a non-polarized splitter (BS). To monitor the beam quality, the reference sub-beam was inspected by a beam profiler. The measurement sub-beam was collected by a dispersive lens L4 (GCL-010158A, Daheng New Epoch Technology, Inc., Beijing, China), whose physical parameters are shown in Table 1, and received by a fiber detector which was mainly composed of a single-mode optical fiber connected to a spectrometer (LEDPRO-50, Ocean Optics, Shanghai, China). A piezo actuator (P843-20, Physik Instrumente (PI) GmbH & Co. KG., Karlsruhe, Germany) with a measurement range of 20 μm and a positioning resolution of 0.6 nm and a linear stage (M-112-1DG, Physik Instrumente (PI) GmbH & Co. KG.) with a measurement range of 25 mm and a positioning resolution of 50 nm were utilized for *X*- and *Z*-axis precision movement of the single-mode optical fiber. Both the *X*- and *Z*-axis moving distances were calibrated by the capacitance probes (ASP-125M-ILA, MTI Instruments Inc., Albany, NY, USA) with a measurement range and resolution of 125 μm and 7.5 nm, respectively.

## 4. Results and Discussions

### 4.1. Measurement Ranges of the X- and Z-Axis

Experiments were first carried out to investigate the FWHM variation with the varying *Z*-axis displacement of the fiber detector and the linear relationship between the FWHM and the *X*-axis displacement within a specific *Z*-axis displacement range. Based on this, the measurement range of the *X*-axis could finally be observed. In these experiments, the entrance face of the single-mode optical fiber core of the fiber detector was mounted onto the linear stage and was made to have a *Z*-directional scanning with a moving step of 1 μm within the *Z*-axis displacement range from 30 to 45 μm. Then, the entrance face of the single-mode optical fiber of the fiber detector was made to move along *X*-axis with a moving interval of 100 nm by the piezo actuator at the *Z*-axis displacements of 30, 35, 40, and 45 μm, respectively. Both the compact linear stage and the piezo actuator were calibrated by the capacitance probes with a measurement range and a measurement resolution of 125 and 7.5 nm, respectively.

**Figure 8:**
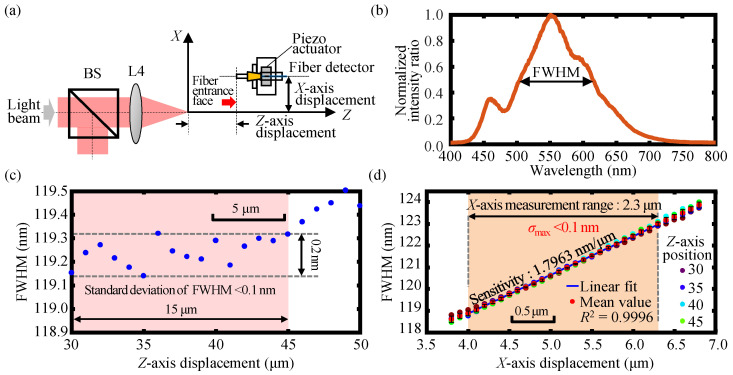
Definition (**a**) of the *X*-axis and *Z*-axis coordinates and the experimental results of (**b**) the obtained output spectrum by the fiber detector and (**c**) the variation of the FWHM within a *Z*-axis displacement range of 15 μm together with (**d**) the linear decoding of FWHM-*X* curves in the corresponding *Z*-axis displacement range of 15 μm.

Figure 8a shows the schematic of the definition of the *X*- and *Z*-axis coordinates in which the focal point of the dispersive lens L4 was set to be the coordinate origin. Figure 8b depicts the detected output spectrum by the fiber detector. Figure 8c,d show the experimental results of the variation of the FWHM within a *Z*-axis displacement range of 15 μm and the linear decoding of FWHM-*X* curves within the corresponding *Z*-axis displacement range. It can be seen from the figures that the change and the standard deviation of the FWHM were less than 0.2 nm and 0.1 nm, respectively, and furthermore, the maximum standard deviation of FWHM at the same *X*-axis displacement *σ*_max_ was less than 0.1 nm within the *Z*-axis displacement range of 15 μm. The experimental results demonstrate that FWHM remained almost constant with the varying of the *Z*-axis displacement of the fiber detector, and furthermore, the linear decoding of the FWHM-*X* curve could be used for indicating the *X*-axis displacement regardless of the simultaneous variation of the *Z*-axis displacement and the *X*-axis measurement range was 2.3 μm. It should be noted that Figure 8d was only a rough estimated *X*-axis displacement measurement range associated with the FWHM variation determined by the *Z*-axis displacement range shown in Figure 8c. It should also be pointed out that the coefficient of determination *R*^2^ was only utilized for a qualitative evaluation of the performance of data fitting, but not as the indicator for the quantitative evaluation of the measurement linearity of the proposed fiber-based chromatic dispersion probe both in simulation and experimental results. Accurate evaluation of the measurement range and measurement linearity with accurate identification of the measurement accuracy and uncertainties will be carried out in future work.

**Figure 9:**
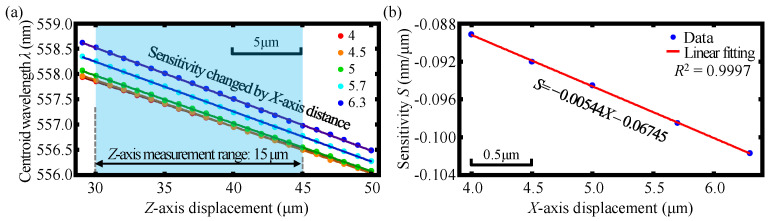
Experimental results of (**a**) the linear relationship between the centroid wavelength *λ* and *Z*-axis displacement and (**b**) the linear relationship between the corresponding sensitivity *S* (*λ-Z* curve slope) and *X*-axis displacement.

Figure 9a depicts the experimental result of the linear relationship between the centroid wavelength *λ* and *Z*-axis displacement. Figure 9b shows the linear relationship between the corresponding sensitivity *S* (*λ-Z* curve slope) and *X*-axis displacement within the *X*-axis displacements from 4 μm to 6.3 μm. As shown in the figures, the centroid wavelength *λ* of the detected spectral signal could be employed for indicating the *Z*-axis displacement associated with different *X*-axis displacements by using the corresponding linear decoding of the *S-X* curve, and furthermore, the *Z*-axis measurement range was 15 μm. It should be pointed out that the output spectra have gradually become spectral bimodal distribution when the *X*-axis displacement was larger than two microns based on the theory of the employed step-index single-mode fiber in which the cladding of the step-index single-mode fiber could hardly accept the dispersive light coupling into it. Based on this, Gaussian fitting was used for the calculation of the centroid wavelength in the simulation. Meanwhile, in practical experiments, not only the core but also the cladding of the step-index single-mode fiber could receive the dispersive light coupling into it; moreover, the focused beam spot diameter by the dispersive lens L4 in the experiment was larger than that of the focused beam spot diameter in simulation due to the influence of the beam quality and the lens spherical aberration. Consequently, no spectral bimodal distribution was found in the practical experiments. Therefore, the simulation and experimental results were different because the output spectrum with Gaussian fitting was used for the calculation of the centroid wavelength in the simulation, which finally resulted in a slight difference in the simulation and experimental results of the equations of the *S-X* decoding. Despite the slight difference, the same trend of the linear relationship could be observed and the difference was, thereby, within the expected tolerance, which demonstrated that the *Z*-axis displacement could be measured regardless of the *X*-axis movement.

### 4.2. Measurement Resolutions of X- and Z-Axis

Experiments were then carried out to investigate the measurement resolutions of the *X*- and *Z*-axis of the established chromatic dispersion probe, respectively. Similarly, in these experiments, the entrance face of the optical fiber core in the fiber detector was fixed onto the piezo actuator and the linear stage was firstly made to have a scanning along *X*-axis with much smaller moving steps of 100, 50, 25, and 15 nm, respectively. Then, the entrance face of the single-mode optical fiber core of the fiber detector was made to move along *Z*-axis with moving steps of 200, 100, 50, and 25 nm, respectively by the linear stage.

**Figure 10:**
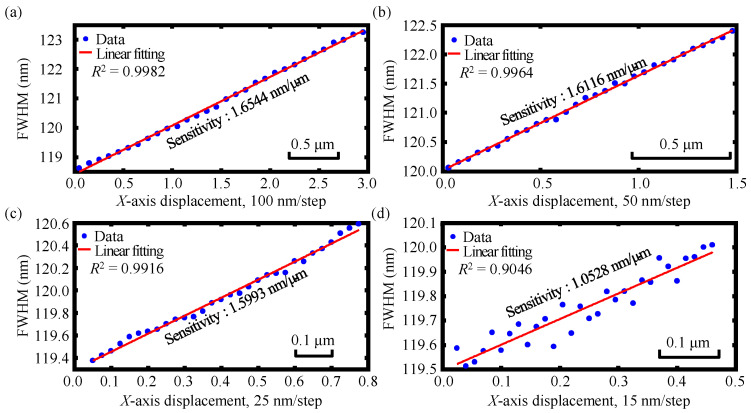
Experimental results of the *X*-axis displacement resolution of the established chromatic dispersion probe with different moving steps of (**a**) 100 nm, (**b**) 50 nm, (**c**) 25 nm, and (**d**) 15 nm, respectively.

**Figure 11:**
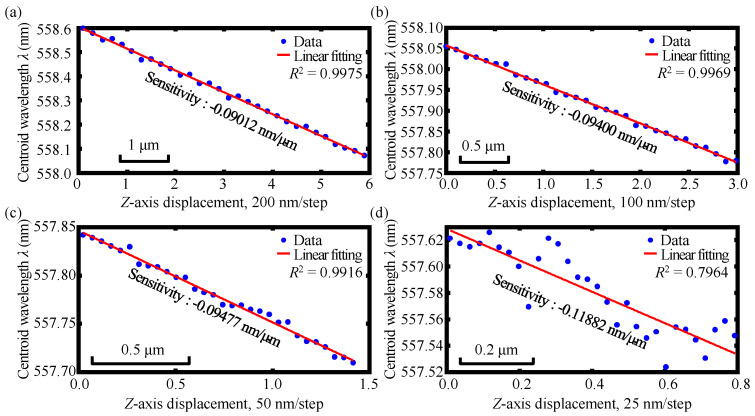
Experimental results of the *Z*-axis displacement resolution of the established chromatic dispersion probe with different moving steps of (**a**) 200 nm, (**b**) 100 nm, (**c**) 50 nm, and (**d**) 25 nm, respectively.

Figure 10 shows the experimental results of the *X*-axis displacement resolution of the established chromatic dispersion probe with the moving steps of 100, 50, 25, and 15 nm, respectively. As can be seen in the figures, the applied moving steps could be clearly discriminated in the moving steps of 100, 50, and 25 nm, and hence, we can conclude that an *X*-axis displacement resolution of better than 25 nm could be realized in the established chromatic dispersion probe. Similarly, Figure 11 depicts the experimental results of the *Z*-axis displacement resolution of the established chromatic dispersion probe with different moving intervals of 200, 100, 50, and 25 nm, respectively, which yielded a *Z*-axis displacement resolution of better than 50 nm for the established dispersion probe. It should be noted that to improve the measurement resolution of the *X*-axis displacement, the sum of sines function was used to fit the detected spectra. As can be seen in Figure 8, Figure 10 and Figure 11, although different sensitivities were found, only slight change could be observed and good measurement linearity could be inspected both in *X*- and *Z*-axis measurement range and resolution experiments. Thus, it could still be considered that the sensitivity was a constant and was independent of the displacement values and measurement linearity within the measurement range of the employed chromatic dispersion probe.

### 4.3. Evaluation of the Basic Performance of the Established Prototype Probe

**Figure 12:**
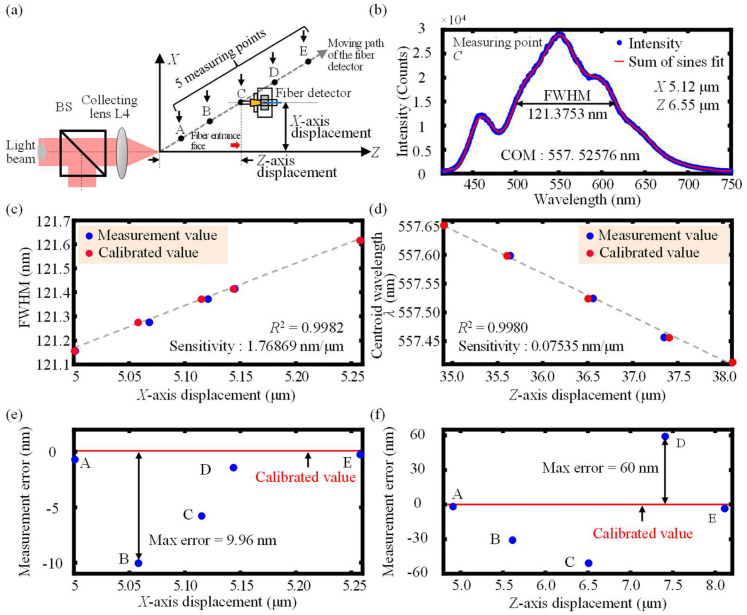
Schematic of (**a**) the moving path of the fiber detector and five randomly selected measuring points A to E. (**b**) Experimental results of the obtained output spectrum of the measuring points C and Experimental results of the simultaneous measurement of (**c**) *X*- and (**d**) *Z*-axis displacements together with the corresponding measurement errors of (**e**) *X*-axis and (**f**) *Z*-axis for the prototype probe.

To verify and confirm the feasibility of the proposed chromatic dispersion probe for the simultaneous measurement of *X*- and *Z*-axis displacements, experiments were finally carried out to evaluate the basic performance of the established prototype probe. In the experiments, as shown in Figure 12a, from the coordinate origin defined in Figure 8a, the moving path of the fiber detector was depicted with *X*- and *Z*-axis moving steps of 100 nm and 1 μm, respectively, along which five measuring points, A to E, were randomly selected. The actual moving displacement was calibrated by the capacitive probe and was called the calibrated value. The displacement decoded from the established chromatic confocal probe was called the measurement value. Figure 12b depicts the experimentally obtained spectrum at the measuring point C and the sum of sines function was used to fit the detected spectrum for the improvement of the measurement resolution of the *X*-axis. Figure 12c,d show the calibrated values (red points) and the measurement values (blue points) for the confirmation experiments of the simultaneous measurement of *X*- and *Z*-axis displacements measurement with nanometric resolutions. As depicted in Figure 12d,e, the maximum measurement errors of the prototype probe were less than 10 nm and 60 nm for *X*- and *Z*-axis displacements measurement, respectively. As can be observed in the experiments, the feasibility of the proposed chromatic dispersion probe for simultaneous measurement of *X*- and *Z*-axis displacements with nanometric resolutions could be verified and simultaneous measurement of *X*- and *Z*-axis displacements of an optical fiber could be realized regardless of the variation of *X*- and *Z*-axis displacements of the optical fiber during the simultaneous measurement process, which greatly fulfills the development of the application of simultaneous measurement of dual-axis displacement in the space-limited and small-cavity fields with nanometric resolutions, in which the micro-sized optical fiber can easily be fixed on a micro-object to be measured.

## 5. Conclusions

In this study, a fiber-based chromatic dispersion probe for the simultaneous measurement of *X*- and *Z*-axis displacements with nanometric resolutions is proposed and demonstrated by using the FWHM of the detected spectral signal, which successfully realized the *X*-axis measurement range and resolution of 2.3 μm and 25 nm, and furthermore, the *Z*-axis measurement range and resolution of 15 μm and 50 nm, respectively. Experiments were performed to evaluate the basic performance of the prototype sensor, which verified the feasibility of the proposed chromatic dispersion probe and yielded that the maximum measurement errors of the probe were less than 10 nm and 60 nm for the *X*- and *Z*-axis displacements, respectively. Due to the micro-size of the optical fiber, it is expected to extend this probe to online calibration in space-limited situations and to enlighten the application in microscale sensing under small-cavity conditions. It should be pointed out that this paper is an initial report of our research on the proposal of a preliminary idea of a fiber-based chromatic dispersion probe for simultaneous measurement of *X*- and *Z*-axis displacements by employing the FWHM of the detected spectrum.

As the next step of our research, studies will be carried out to systematically optimize the proposed fiber-based chromatic dispersion probe and achieve longer measurement ranges together with higher measurement resolutions for both the *X*- and *Z*-axis. Moreover, the practical application of the developed fiber-based chromatic dispersion probe to the dual-axis displacement measurement under space-limited and small-cavity conditions in the industry remains a challenge to be addressed and will also be taken into consideration in the next step of our research.

## Figures and Tables

**Table 1:** Physical parameters of the dispersive lens.

Items	Symbol	Value
Lens materials	-	K9
Surface radii of the lens L4	*R*_1_	∞
	*R*_2_	15.5 mm
Sellmeier coefficients	*B*_1_	1.03961212
	*B*_2_	0.231792344
	*B*_3_	1.01046945
	*C*_1_	0.00600069867
	*C*_2_	0.0200179144
	*C*_3_	103.560653

## Data Availability

Not applicable.

## References

[1] Baier V., Schardt M., Fink M., Jakobi M., Koch A.W. (2022). MEMS-Scanner Testbench for High Field of View LiDAR Applications. Sensors.

[2] Li H., Ma X., Cui B., Wang Y., Zhang C., Zhao J., Zhang Z., Tang C., Li E. (2017). Chip-scale demonstration of hybrid III–V/silicon photonic integration for an FBG interrogator. Optica.

[3] Liu S., Chen X., Zhang C. (2015). Development of a broadband Mueller matrix ellipsometer as a powerful tool for nanostructure metrology. Thin Solid Films.

[4] Wu W., Jin G., Zhu J. (2019). Optical design of the freeform reflective imaging system with wide rectangular FOV and low F-number. Results Phys..

[5] Zhao W., Sun Y., Wang Y., Qiu L., Shao R., Cui H. (2018). Three-dimensional super-resolution correlation-differential confocal microscopy with nanometer axial focusing accuracy. Opt. Express.

[6] Zhu J., Cui P., Guo Y., Yang L., Lin J. (2015). Pulse-to-pulse alignment based on interference fringes and the second-order temporal coherence function of optical frequency combs for distance measurement. Opt. Express.

[7] Brown J.J., Dikin D.A., Ruoff R.S., Bright V.M. (2012). Interchangeable Stage and Probe Mechanisms for Microscale Universal Mechanical Tester. J. Microelectromech. Syst..

[8] Yang M.-Z., Dai C.-L., Lu D.-H. (2010). Polypyrrole Porous Micro Humidity Sensor Integrated with a Ring Oscillator Circuit on Chip. Sensors.

[9] Bristow D.A., Alleyne A. (2006). A High Precision Motion Control System With Application to Microscale Robotic Deposition. IEEE Trans. Control Syst. Technol..

[10] McDaniel G.W., McKelvey K.S. (2006). A Cost-Effective System for Measuring Microscale Habitat Use of Small Mammals with High Precision. Wildl. Soc. Bull..

[11] Cai Y., Xie B., Wen Z., Fan K.-C. (2021). A miniature laser diode interferometer with self-compensation of retroreflector’s motion errors for displacement feedback of small-sized micro/nano motion stages. Measurement.

[12] Lu S., Yan P., Zhang B. (2020). Long stroke displacement measurement with reduced coupling error supporting high precision control of a beam flexure-based micro-stage. Rev. Sci. Instrum..

[13] Kim Y.-S., Dagalakis N.G., Choi Y.-M. (2018). Optical fiber Fabry-Pérot micro-displacement sensor for MEMS in-plane motion stage. Microelectron. Eng..

[14] Li H., Zhu B., Chen Z., Zhang X. (2019). Realtime in-plane displacements tracking of the precision positioning stage based on computer micro-vision. Mech. Syst. Signal Process..

[15] Ya’akobovitz A., Krylov S., Hanein Y. (2010). Nanoscale displacement measurement of electrostatically actuated micro-devices using optical microscopy and digital image correlation. Sens. Actuators A Phys..

[16] Peng Y., Ito S., Shimizu Y., Azuma T., Gao W., Niwa E. (2014). A Cr-N thin film displacement sensor for precision positioning of a micro-stage. Sens. Actuators A Phys..

[17] Adachi K., Matsukuma H., Sugawara T., Shimizu Y., Gao W., Niwa E., Sasaki Y. (2019). Integration of a Cr–N Thin-Film Displacement Sensor into an XY Micro-stage for Closed-Loop Nano-positioning. Nanomanuf. Metrol..

[18] Xu H.G., Ono T., Esashi M. (2006). Precise motion control of a nanopositioning PZT microstage using integrated capacitive displacement sensors. J. Micromechanics Microeng..

[19] Ji L., Zhu Y., Moheimani S.O.R., Yuce M.R. A micromachined 2DOF nanopositioner with integrated capacitive displacement sensor. Proceedings of the SENSORS, 2010 IEEE.

[20] Yang S., Huang C. (2009). A Hall Sensor-Based Three-Dimensional Displacement Measurement System for Miniature Magnetically Levitated Rotor. IEEE Sens. J..

[21] Gao W., Saito Y., Muto H., Arai Y., Shimizu Y. (2011). A three-axis autocollimator for detection of angular error motions of a precision stage. CIRP Ann..

[22] Gao W., Kimura A. (2007). A Three-axis Displacement Sensor with Nanometric Resolution. CIRP Ann..

[23] Mirko H., Jörg R. (2015). Error motion compensating tracking interferometer for the position measurement of objects with rotational degree of freedom. Opt. Eng..

[24] Ren M., Liang J., Li L., Wei B., Wang L., Tang Z. (2015). Accurate three-dimensional shape and deformation measurement at microscale using digital image correlation. Rev. Sci. Instrum..

[25] Khorshad A.A., Hassani K., Tavassoly M.T. (2012). Nanometer displacement measurement using Fresnel diffraction. Appl. Opt..

[26] Wang X., Guo X., Wang Y., Jiang C., Jiang J., Zhang Z. (2020). All-fiber differential interferometer for nanometric displacement measurement. Opt. Commun..

[27] Cazacu S., Martins J.M., Rego G., Santos S.F., Santos J.L., Baptista J.M. (2004). Micro-displacement measurement using a long period fiber grating in a self-referenced fiber optic intensity sensor. Proceedings of the 17th Annual Meeting of the IEEE Lasers and Electro-Optics Society, 2004, LEOS 2004.

[28] Rahman H.A., Harun S.W., Yasin M., Ahmad H. (2012). Fiber-Optic Salinity Sensor Using Fiber-Optic Displacement Measurement With Flat and Concave Mirror. IEEE J. Sel. Top. Quantum Electron..

[29] Puangmali P., Althoefer K., Seneviratne L.D. (2010). Mathematical Modeling of Intensity-Modulated Bent-Tip Optical Fiber Displacement Sensors. IEEE Trans. Instrum. Meas..

[30] Lin D., Jiang X., Xie F., Zhang W., Zhang L., Bennion I. (2004). High stability multiplexed fibre interferometer and its application on absolute displacement measurement and on-line surface metrology. Opt. Express.

[31] Suganuma F., Shimamoto A., Tanaka K. (1999). Development of a differential optical-fiber displacement sensor. Appl. Opt..

[32] Ghaffar A., Hou Y.-L., Liu W.-Y., Dharejo F.A., Zhang H.-X., Jia P., Yanyun H., Liu J., Yunjun Z., Nasir Z. (2019). Two-dimensional displacement optical fiber sensor based on macro-bending effect. Opt. Laser Technol..

[33] Qi L., Zhao C.-L., Wang Y., Kang J., Zhang Z., Jin S. (2013). Compact micro-displacement sensor with high sensitivity based on a long-period fiber grating with an air-cavity. Opt. Express.

[34] Bravo M., Pinto A.M.R., Lopez-Amo M., Kobelke J., Schuster K. (2012). High precision micro-displacement fiber sensor through a suspended-core Sagnac interferometer. Opt. Lett..

[35] Chen C., Shimizu Y., Sato R., Matsukuma H., Gao W. (2020). An off-axis differential method for improvement of a femtosecond laser differential chromatic confocal probe. Appl. Sci..

[36] Mizutani Y., Groves R.M. (2011). Multi-Functional Measurement Using a Single FBG Sensor. Exp. Mech..

[37] Ocvirk G., Tang T., Jed Harrison D. (1998). Optimization of confocal epifluorescence microscopy for microchip-based miniaturized total analysis systems. Analyst.

[38] Kimura S., Wilson T. (1991). Confocal scanning optical microscope using single-mode fiber for signal detection. Appl. Opt..

[39] Goodman J.W. (1968). Introduction to Fourier Optics.

[40] Barrell K.F., Pask C. (1979). Optical Fibre Excitation by Lenses. Opt. Acta: Int. J. Opt..

[41] Snyder A.W. (1969). Asymptotic Expressions for Eigenfunctions and Eigenvalues of a Dielectric or Optical Waveguide. IEEE Trans. Microw. Theory Tech..

[42] Snyder A., Love J. (1983). Optical Waveguide Theory.

